# Identification of early prediction biomarkers of severity in patients with severe fever with thrombocytopenia syndrome based on plasma proteomics

**DOI:** 10.3389/fmicb.2025.1514388

**Published:** 2025-02-05

**Authors:** Qian Zhang, Zhengyi Jiang, Nan Jiang, Luchen Shi, Jiaying Zhao, Jie Zhao, Ke Ouyang, Huaying Huang, Yaqin Zhang, Yan Dai, Nannan Hu, Ping Shi, Yaping Han, Ke Jin, Jun Li

**Affiliations:** ^1^Department of Infectious Disease, The First Affiliated Hospital of Nanjing Medical University, Nanjing, China; ^2^Department of Infectious Disease, Shanghai Ninth People’s Hospital, Shanghai Jiao Tong University School of Medicine, Shanghai, China; ^3^Department of Respiratory Disease, Yixing No. 2 People’s Hospital, Yixing, China; ^4^Department of Infectious Disease, Nanjing Second Hospital, Nanjing University of Chinese Medicine, Nanjing, China; ^5^School of Integrated Chinese and Western Medicine, Nanjing University of Chinese Medicine, Nanjing, China

**Keywords:** severe fever with thrombocytopenia syndrome, proteomics, biomarker, prediction, prognosis

## Abstract

**Background:**

Severe fever with thrombocytopenia syndrome (SFTS) is a newly emerging infectious disease. Given its rapid disease progression and high mortality rate, early warning is crucial in improving the outcomes, However, to date, relevant comprehensive predictors or an effective prediction model are still poorly explored.

**Methods:**

A plasma proteomic profile was performed at early stages in patients with SFTS. Functional clustering analysis was used to select the candidate proteins and then validate their expression by ELISA. A cohort consisting of 190 patients with SFTS was used to develop the predictive model for severe illness and subsequently validate it in a new cohort consisting of 93 patients with SFTS.

**Results:**

A significant increase in plasma proteins associated with various functional clusters, such as the proteasomal protein catabolic process, phagocytosis, and humoral immune response, was observed in severe SFTS patients. High levels of four proteins including NID1, HSP90α, PSMA1, and VCAM1 were strongly correlated with multi-organ damage and disease progression. A prediction model was developed at the early stage to accurately predict severe conditions with the area under the curve of 0.931 (95% CI, 0.885, 0.963).

**Conclusion:**

The proteomic signatures identified in this study provide insights into the potential pathogenesis of SFTS. The predictive models have substantial clinical implications for the early identification of SFTS patients who may progress to severe conditions.

## Introduction

Severe fever with thrombocytopenia syndrome (SFTS) is a newly emerging infectious disease first reported in China in 2009 (Yu et al., 2011), with a 5–45% fatality rate (Cui et al., 2015; Chen et al., 2022b). The causative agent was identified as a novel *Phlebovirus* in the *Bunyaviridae* family, previously named SFTS virus, newly named *Dabie bandavirus* (DBV) (Walker et al., 2019). SFTS was subsequently reported in other Asian countries, including South Korea, Japan, Vietnam, Myanmar, and Thailand (Nam et al., 2023). DBV is primarily transmitted by tick-to-human, and can also be transmitted from person to person via contact with infected blood (Chen et al., 2022a). Because of its high case fatality rate, multiple transmission routes, and extensive geographical distribution, SFTS has become a significant global infectious disease.

SFTS manifests with abrupt onset of high fever, lymphadenopathy, and respiratory or gastrointestinal symptoms. Severe cases might develop encephalopathy, hemorrhaging, sepsis, and multiple organ failure (Li H. et al., 2018). Laboratory extreme abnormalities include progressive thrombocytopenia and leukopenia and elevated serum levels of aspartate aminotransferase (AST), lactate dehydrogenase (LDH), creatine kinase (CK), and serum creatinine (sCr) (Yu, 2018; Zhao et al., 2022). Hyper-viremia-induced pathophysiological changes such as cytokine storm, reduction and functional impairment of immune cells, endothelial damage as well as disseminated intravascular coagulation played important roles in the disease progress (Peng et al., 2016; Yamada et al., 2018; Wang Y. N. et al., 2022). It is imperative to identify predictors associated with disease progression for optimal clinical management. However, to date, relevant comprehensive predictors or effective prediction models are still poorly explored.

Alterations of patients’ plasma proteins are informative and have been well-recognized as indicators of pathophysiological changes during the disease progression in various epidemic infectious diseases, including coronavirus disease 2019 (Shu et al., 2020), severe acute respiratory syndrome (Ren et al., 2004), and Zika virus infection (Wee et al., 2019). Similarly, serum proteomic changes related to pathophysiological changes such as thrombocytopenia, abnormal immune response, and inflammatory activation were documented in SFTS patients (Lee et al., 2022). Some proteins were identified as biomarkers for predicting SFTS prognosis (Zhang et al., 2024). Therefore, to find more accurate biomarkers for disease progression of SFTS, we performed quantitative proteomics analysis to identify several early proteins that displayed significant alterations in the plasma of severe SFTS patients. The plasma proteins were further validated by enzyme-linked immunosorbent assay (ELISA) in a large-scale cohort. Furthermore, we developed a combination of biomarkers based on these proteins that could accurately predict the patients who are at risk of developing severe conditions. These findings provided valuable knowledge about plasma biomarkers correlated with disease progression in SFTS, potentially shedding light on the underlying pathogenesis of SFTS and facilitating early identification of severe cases to enhance disease prognosis.

## Methods

### Study design and patients

**Figure 1:**
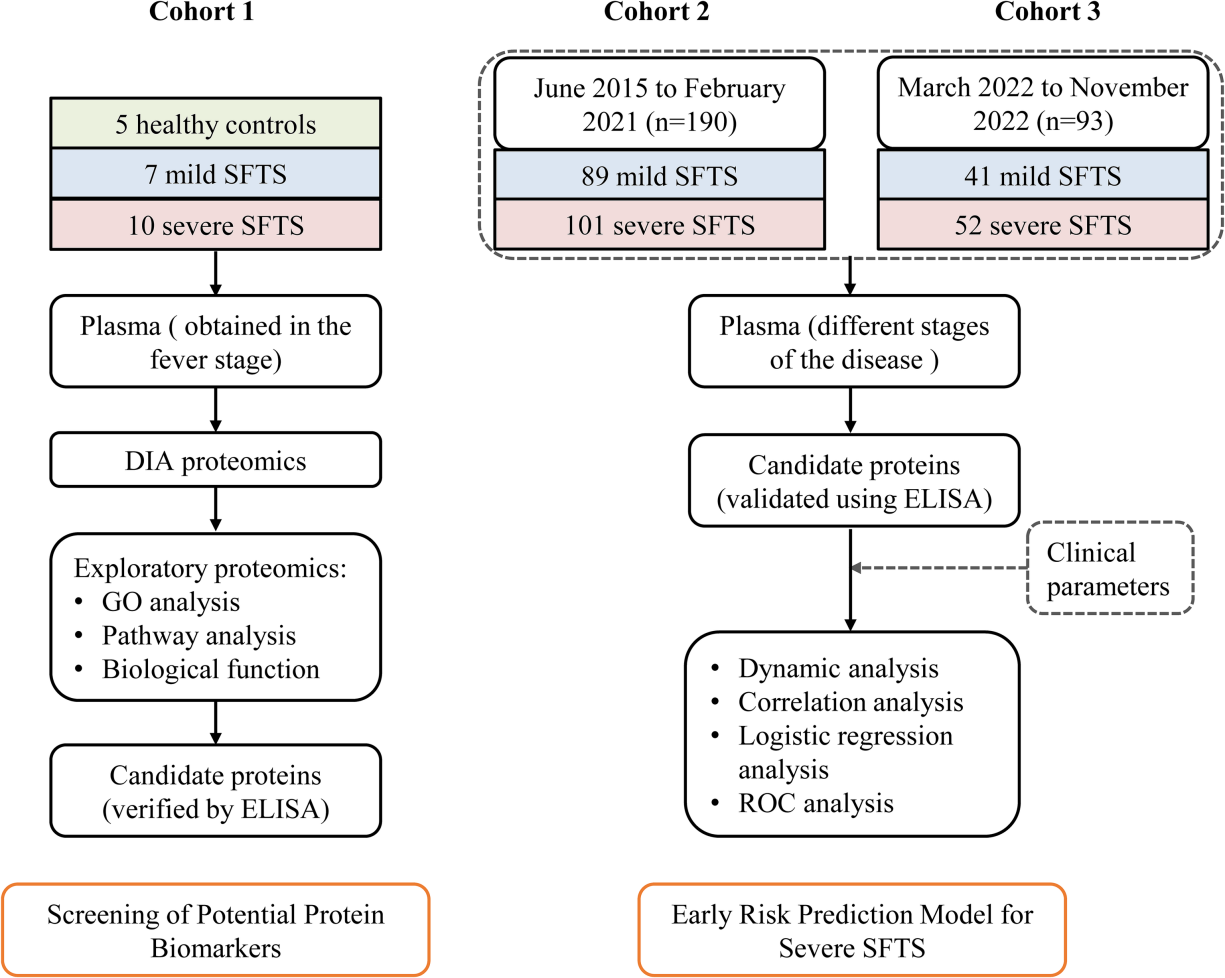
Study design overview for severe SFTS prediction model development. Cohort 1, including 5 healthy controls, 7 mild SFTS patients and 10 severe patients, was used to identify potential plasma protein biomarkers associated with severe illness. Cohort 2, composed of 89 mild SFTS and 101 severe patients, was used to establish a prediction model for severe SFTS based on these biomarkers and clinical parameters. Cohort 3, consisting of 41 mild SFTS and 52 severe SFTS, was used to validate the efficacy of the prediction model.

We performed quantitative proteomics in plasma samples from 7 mild (M) and 10 severe (S) cases who were diagnosed with SFTS at the First Affiliated Hospital of Nanjing Medical University from April 2021 to July 2021. Five age- and gender-matched healthy (H) subjects were enrolled as controls (Cohort 1). To develop a better early severe prediction model, a total of 190 SFTS patients were enrolled in the same center from June 2015 to February 2021 (Cohort 2). Additionally, to validate its efficacy, 93 SFTS patients were prospectively recruited from March 2022 to November 2022 (Cohort 3). Plasma samples were obtained from all patients during the fever stage at the time of enrollment (Figure 1). Among the patients, plasma samples were collected from 86 cases at different stages of the disease for dynamic analysis of protein markers. In addition, 20 healthy subjects, whose serological tests were negative for DBV, were enrolled for comparison.

Diagnostic criteria of SFTS were as follows: (1) possibility of tick bite history; (2) acute fever, fatigue with thrombocytopenia; and (3) positive DBV ribonucleic acid (RNA) confirmed by real-time quantitative reverse-transcription polymerase chain reaction in plasma.

According to previous reports (Chen G. et al., 2022; Li and Wang, 2022), cases that met any one of the following criteria were classified as severe: (1) presenting with severe neurological symptoms, (2) multiple organ failure, (3) severe bleeding, (4) severe secondary infection, (5) platelet count <30 × 10^9^/L, (6) AST, LDH, CK >10 × ULN (upper limit of normal) or (7) death. Exclusive criteria were as follows: (1) laboratory-confirmed other pathogen infections, (2) underlying serious systemic diseases, or (3) severe immune deficiency. Demographic and laboratory data were obtained from all the patients. The survival status of all patients was followed for 28 days post-disease onset.

Written informed consent was obtained from each patient following the principles of the Declaration of Helsinki. The study was approved by the Research and Ethics Committee of the First Affiliated Hospital of Nanjing Medical University, Nanjing, China (Ethics approval number, 2022-SR-366).

### Collection of plasma samples

Blood samples from all patients were collected in K2-EDTA tubes at the indicated time points. The blood was processed immediately to centrifuge at 670 g for 10 min at 20°C and the plasma was aliquoted and stored at −80°C until at the time of assays.

### Dabie banda-virus detection

Viral RNA was extracted from plasma samples with a commercial kit (Daan Gene, Guangzhou, China) by a trained specialist. The extracted RNA was amplified using specific primers and probes by one-step real-time fluorescence qRT-PCR for the detection of a novel bunyavirus in all specimens according to the manufacturer’s instructions. Real-time qRT-PCR cycling was performed as follows: after reverse transcription at 50°C for 15 min, polymerase was activated at 95°C for 15 min, and amplification was undertaken for 45 cycles consisting of a denaturing step at 94°C for 15 s and an annealing-extension step at 55°C for 45 s. The fluorogenic signal emitted was collected during the extension step, and a cycle threshold was acquired for quantifying the viral load of the sample.

### LC-MS/MS and data analysis

The samples were fractionated using a high pH reversed-phase fractionator, peptide fractions were subsequently analyzed by LC-MS/MS using the Thermo Scientific Orbitrap Exploris^™^ 480 platform (Thermo Fisher Scientific) via data-independent acquisition (DIA) scan mode. Mass spectrometry data were used to generate a hybrid library in the Spectronaut software (Biognosys, version 15.7) for final protein identification and quantitation. All searches were performed against the human UniProt proteome sequences, LianChuan Biotechnology Company (Hangzhou, China) provides related technical support. Detailed procedures can be found in the Supplementary material.

### Functional clustering analysis

Acquired data were analyzed by Excel 2021 and R (Version 4.2.0). Proteins with missing values in more than 60% of samples were excluded. The remaining missing values were interpolated by the median. Principal component analysis (PCA) and Clustering heatmaps of proteins using “ggplot2” and “pheatmap” packages, respectively. Differentially expressed proteins (DEPs) were defined as average ratio-fold change (FC) >1.5 as well as *p*-value <0.05. The DEPs were subjected to Gene Ontology (GO) functional clustering analyses.

### Enzyme-linked immunosorbent assay

Human protein ELISA kits were used to quantify plasma levels of proteins according to manufacturers’ instructions. Briefly, plasma samples were diluted according to the manufacturer’s dilution guidelines. Then, 100 μL of fixed dilution plasma sample was added to the plates and incubated for 90 min at 37°C. After washing, 100 μL biotinylated-specific antibody was immediately added to each well, and the plates were incubated for 60 min at 37°C. Followed by washing, 100 μL Avidin-horseradish peroxidase was added and incubated for 30 min at 37°C. Finally, the optical density (OD) value at 450 nm was determined after the addition of 90 μL tetramethyl-benzidine reagent and stop solution. The standard curve of each protein was generated by the determination of OD values from serial dilutions of the standard samples with known protein concentrations. Detailed information on ELISA kits can be found in Supplementary Table 1.

### Statistical analysis

Statistical analyses were performed using SPSS 25.0 (IBM, Armonk, NY) or otherwise specified. Values are expressed as the mean ± standard deviation for normally distributed or as the median (*p*_25_, *p*_75_) for nonnormally distributed, whereas categorical variables are expressed as percentages. The independent Student *t*-test, nonparametric Mann–Whitney *U* test, or *χ*^2^ test was used to determine differences among groups. Correlations were analyzed using Spearman’s correlation analysis. Logistic regression analysis was used to identify variables for predicting severe SFTS patients. The area under the receiver operating characteristic curve (auROC) was calculated and compared by the *Z* test (Delong’s method). Differences with a *p* < 0.05 were considered statistically significant.

## Results

### Identification of potential early-stage protein biomarkers for SFTS

**Figure 2:**
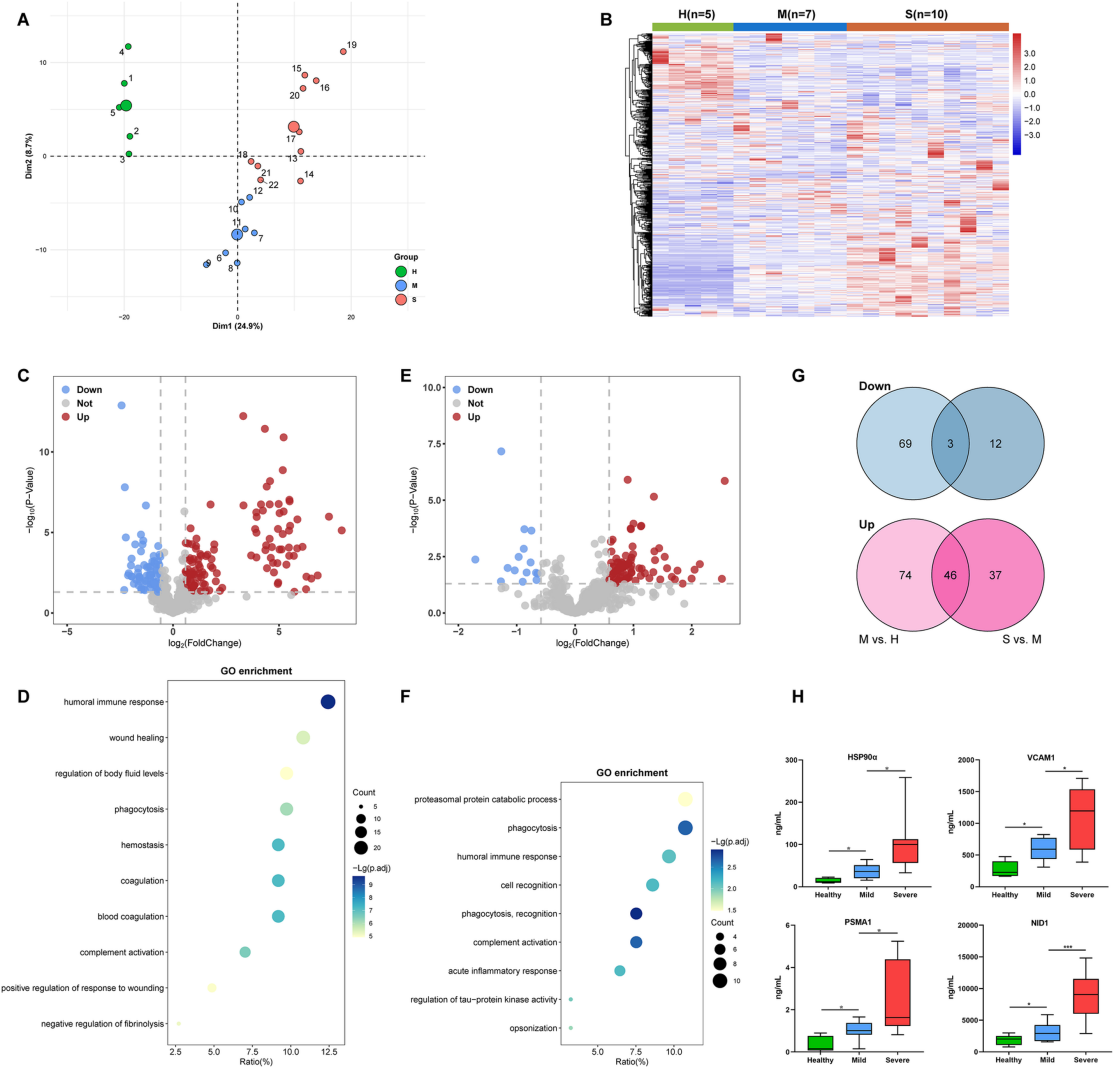
Identification of candidate proteins associated with severe SFTS patients in Cohort 1. **(A)** PCA plot of the proteomics data (583 quantified proteins). Green, blue, and red points represent healthy control, mild, and severe SFTS patients, respectively. The subject ID numbers were also displayed. The centroid of each sample group was marked by a larger solid circle. **(B)** A clustering heatmap of the protein expression levels. Red colors represent high expression levels and blue colors represent low expression levels. The color intensity was proportional to the expression levels. H, healthy control; M, mild cases; S, severe cases. **(C)** Volcano plot visualizing the identified DEPs of the M group compared with the H group. The red dots represent upregulated DEPs, while the blue dots represent downregulated DEPs (Fold change >1.5 and *p* < 0.05), and the gray dots represent no significance. **(D)** Dot-plot visualization of GO biological process terms for DEPs between the M group versus the H group. The dot size was proportional to the number of proteins annotated to the corresponding GO term. The color-scale was proportional to −log_10_ (adjusted *p*-value). **(E)** Volcano plot visualizing the identified DEPs of the S group compared with the M group. The red dots represent upregulated DEPs, while the blue dots represent downregulated DEPs (Fold change >1.5 and *p* < 0.05), and the gray dots represent no significance. **(F)** Dot-plot visualization of GO biological process terms for DEPs between the S group versus the M group. The dot size was proportional to the number of proteins annotated to the corresponding GO term. The color-scale was proportional to −log_10_ (adjusted *p*-value). **(G)** Venn diagram visualization of DEPs abundance trends among the H, M, and S groups. **(H)** ELISA was performed to validate the expression levels of four candidate proteins (HSP90α, VCAM1, PSMA1, and NID1) in the plasma of subjects from cohort1. *, **, and *** represent *p* < 0.05, *p* < 0.01, and *p* < 0.001, respectively.

To identify the potential protein biomarkers, we performed quantitative proteomics in plasma samples from Cohort 1 (5 H, 7 M, and 10 S). The clinical baseline characteristics of Cohort 1 were described in Table 1. A total of 583 proteins were identified by proteomics analysis. PCA showed that the protein profiles of the severe and mild patients were visibly distinct from the healthy control, and protein distribution in the severe group was segregated from the mild group (Figure 2A). Similarly, the heatmap clustering analysis also demonstrated differences in protein expression profiles among the three groups (Figure 2B), indicating unique proteomic features in severe cases for further exploration.

**Table 1:** Comparison of clinical characteristics in SFTS patients from Cohort 1.

Variables	Healthy controls (*n* = 5)	Mild SFTS (*n* = 7)	Severe SFTS (*n* = 10)	*p*-value (M vs. H)	*p*-value (S vs. M)
Age (year)	65.2 ± 3.6	59.1 ± 8.6	71.7 ± 5.2	0.130	0.002
Male, *n* (%)	2 (40.0)	3 (42.9)	5 (50.0)	1.000	1.000
Hypertension	0 (0.0)	1 (14.3)	3 (30.0)	1.000	0.603
Diabetes	0 (0.0)	0 (0.0)	0 (0.0)	—	—
Complications (%)					
Encephalitis	—	—	4 (40.0)	—	—
Multiple organ failure	—	—	2 (20.0)	—	—
Severe hemorrhage	—	—	0 (0.0)	—	—
Severe infection	—	—	2 (20.0)	—	—
28 days death, *n* (%)	—	0 (0.0)	3 (30.0)	—	—
Laboratory parameters on admission					
PLT (×10^9^/L)	186 ± 27	86 ± 35	47 ± 15	—	—
ALT (U/L)	23 ± 5	47 ± 18	121 ± 88	—	—
AST (U/L)	24 ± 3	79 ± 35	318 ± 306	—	—
LDH (U/L)	199 ± 31	597 ± 374	817 ± 374	—	—
CK (U/L)	102 ± 30	570 ± 569	555 ± 510	—	—

SFTS, severe fever with thrombocytopenia syndrome; H, healthy; M, mild; S, severe; PLT, platelet; ALT, alanine aminotransferase; AST, aspartate aminotransferase; LDH, lactate dehydrogenase; CK, creatine kinase. Data are presented as the mean ± standard deviation, median (*p*_25_, *p*_75_), and proportion.

Next, we picked out several plasma proteins related to severe cases from proteomic results. The volcano plot showed that, Compared to healthy controls, a total of 120 significantly upregulated and 72 significantly downregulated DEPs were identified in mild patients (Figure 2C). GO enrichment analysis showed that DEPs were highly enriched in biological processes involved in humoral immune response, phagocytosis, hemostasis, and coagulation (Figure 2D). Compared to mild patients, a total of 83 significantly upregulated and 15 significantly downregulated DEPs were identified in severe patients (Figure 2E). The GO-terms pathways of the DEPs were highly enriched in processes related to proteasomal protein catabolic process, phagocytosis, and humoral immune response (Figure 2F). We further found that 46 DEPs exhibited an increasing trend, while three DEPs exhibited a decreasing trend among the H, M, and S groups (Figure 2G). Then, based on their abundance and biological functions primarily involved in processes such as inflammation, immunity response, metabolism, or coagulation which were related to pathological and physiological changes in the disease, 18 DEPs were finally selected for validation in plasma using ELISA (Table 2). Finally, consistent with proteomics results, four proteins, namely heat shock protein 90α (HSP90α), vascular cell adhesion protein 1 (VCAM1), proteasome subunit alpha type 1 (PSMA1), and nidogen 1 (NID1) were elevated in the early-stage plasma of severe SFTS patients (Figure 2H). Thus, we choose those four proteins to serve as candidate biomarkers for further research.

**Table 2:** Information of 18-candidate proteins in plasma of SFTS patients.

UniProt_ID	Protein	Proteomics				ELISA			
		FC (M/H)	*p*-value	FC (S/M)	*p*-value	Ratio (M/H)	*p*-value	Ratio (S/M)	*p*-value
Inflammatory reaction									
P08238	HSP90AB1	1.6	3.3 × 10^−3^	1.7	1.0 × 10^−3^	0.7	NS	1.9	NS
P07900	**HSP90α**	44.4	7.7 × 10^−6^	1.7	2.5 × 10^−2^	2.5	2.9 × 10^−2^	2.7	2.2 × 10^−2^
P25786	**PSMA1**	3.9	5.5 × 10^−5^	2.1	3.7 × 10^−2^	2.7	3.7 × 10^−2^	2.5	3.6 × 10^−2^
P19320	**VCAM1**	3.1	2.4 × 10^−4^	1.7	2.8 × 10^−2^	2.1	1.1 × 10^−2^	2.3	2.7 × 10^−2^
Immunological reaction									
P62805	H4C1	2.7	1.5 × 10^−2^	1.9	1.1 × 10^−2^	1.9	NS	1.1	NS
P14543	**NID1**	2.8	4.2 × 10^−3^	1.7	1.2 × 10^−2^	1.6	4.1 × 10^−2^	2.9	2.4 × 10^−4^
P08637	FcγR3A	1.5	2.6 × 10^−3^	1.6	1.4 × 10^−2^	0.5	NS	1.1	NS
P26022	PTX3	44.7	2.8 × 10^−4^	1.6	4.1 × 10^−2^	1.3	4.2 × 10^−2^	1.0	NS
Q08830	FGL1	4.0	1.1 × 10^−2^	1.7	3.9 × 10^−2^	4.0	1.9 × 10^−3^	1.1	NS
Metabolic process									
P28838	LAP3	12.9	2.7 × 10^−4^	1.9	2.9 × 10^−2^	1.1	NS	1.1	NS
P49006	MARCKS	23.6	1.8 × 10^−7^	1.5	5.9 × 10^−3^	0.4	NS	1.0	NS
P80723	BASP1	3.5	4.5 × 10^−4^	1.6	1.7 × 10^−2^	0.8	NS	1.3	NS
P04424	ASL	20.5	1.3 × 10^−4^	3.4	1.3 × 10^−2^	1.0	NS	0.8	NS
P23381	WARS1	37.2	5.0 × 10^−7^	1.7	1.2 × 10^−2^	1.0	NS	1.3	3.0 × 10^−2^
O75874	IDH1	24.4	3.8 × 10^−4^	2.2	1.1 × 10^−2^	1.5	1.4 × 10^−2^	0.9	NS
P25789	PSMA4	34.8	1.1 × 10^−4^	2.6	1.9 × 10^−3^	1.0	NS	1.1	NS
Coagulation cascade									
P05154	PCI	0.3	5.5 × 10^−5^	0.5	4.1 × 10^−2^	1.1	NS	1.3	NS
P02776	PF4	0.4	4.7 × 10^−3^	0.6	1.8 × 10^−2^	1.5	NS	1.9	1.5 × 10^−2^

SFTS, Severe fever with thrombocytopenia syndrome; ELISA, enzyme-linked immunosorbent assay; FC, fold change; H, healthy; M, mild; S, severe; HSP90AB1, Heat shock protein 90AB1; HSP90α, Heat shock protein 90α; PSMA1, Proteasome subunit alpha type 1; VCAM1, Vascular cell adhesion protein 1; H4C1, Histone H4; NID1, Nidogen 1; FcγR3A, Low affinity immunoglobulin gamma Fc region receptor III-A; PTX3, Pentraxin-related protein 3; FGL1, Fibrinogen-like protein 1; LAP3, Cytosol aminopeptidase; MARCKS, Myristoylated alanine-rich C-kinase substrate; BASP1, Brain acid soluble protein 1; ASL, Argininosuccinate lyase; WARS1, Tryptophan-tRNA ligase; IDH1, Isocitrate dehydrogenase cytoplasmic; PSMA4, Proteasome subunit alpha type 4; PCI, Plasma serine protease inhibitor; PF4, Platelet factor 4; NS, no statistical difference. The bold protein indicates those proteins were statistical significant trends among those three groups both in proteomics and ELISA result.

### Candidate proteins correlated with multi-organ impairment and disease progression in patients with SFTS

**Figure 3:**
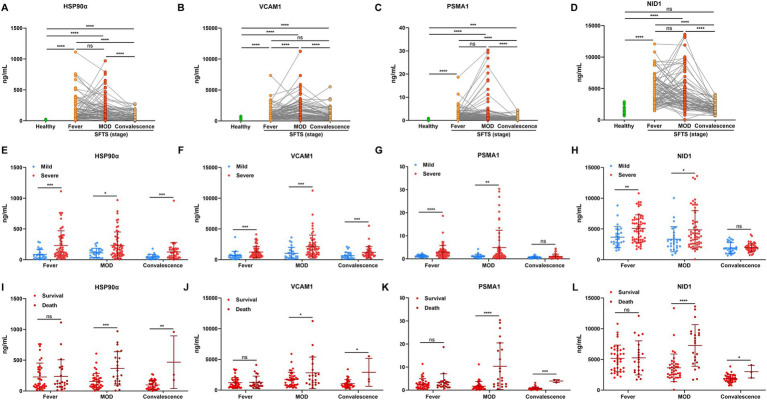
The dynamic alterations of plasma HSP90α, VCAM1, PSMA1, and NID1 levels in patients with SFTS. The concentration of HSP90α **(A)**, VCAM1 **(B)**, PSMA1 **(C)**, and NID1 **(D)** in SFTS patients (*n* = 86) and healthy controls (*n* = 20). The green and red (orange) dots represent the healthy and the SFTS patients, respectively. The expression of HSP90α **(E)**, VCAM1 **(F)**, PSMA1 **(G)**, and NID1 **(H)** in mild SFTS patients (*n* = 26) compared with severe SFTS patients (*n* = 60) SFTS patients. The blue and red dots represent the mild cases and the severe cases, respectively. The levels of HSP90α **(I)**, VCAM1 **(J)**, PSMA1 **(K)** and NID1 **(L)** in survival (*n* = 38) and death outcome (*n* = 22) from severe SFTS patients. The red and dark red dots represent the survivors and the deceased patients, respectively. ns, *, **, ***, and **** represent no statistical difference, *p* < 0.05, *p* < 0.01, *p* < 0.001, and *p* < 0.0001, respectively.

According to the typical clinical features, the course of SFTS has the following phases: the fever stage (1–7 days after onset, early-stage), the MOD stage (8–14 days), and the convalescent stage (>14 days). We analyzed the alternations of candidate protein expression in plasma from 86 cases in these three phases (20 H, 26 M, and 60 S). Results showed that the levels of candidate proteins including HSP90α, VCAM1, PSMA1, and NID1 were higher in the fever stage of SFTS patients compared with healthy controls, almost reaching plateaus in the MOD stage, and the concentrations were decreased in the convalescent stage (Figures 3A–D). Furthermore, the candidate protein levels were all higher in the severe cases compared to the mild cases both in the fever and MOD stages (Figures 3E–H). It was particularly noteworthy that the candidate protein levels were significantly higher in the non-survivors compared to the survivors in both the MOD and convalescence stages (Figures 3I–L).

**Figure 4:**
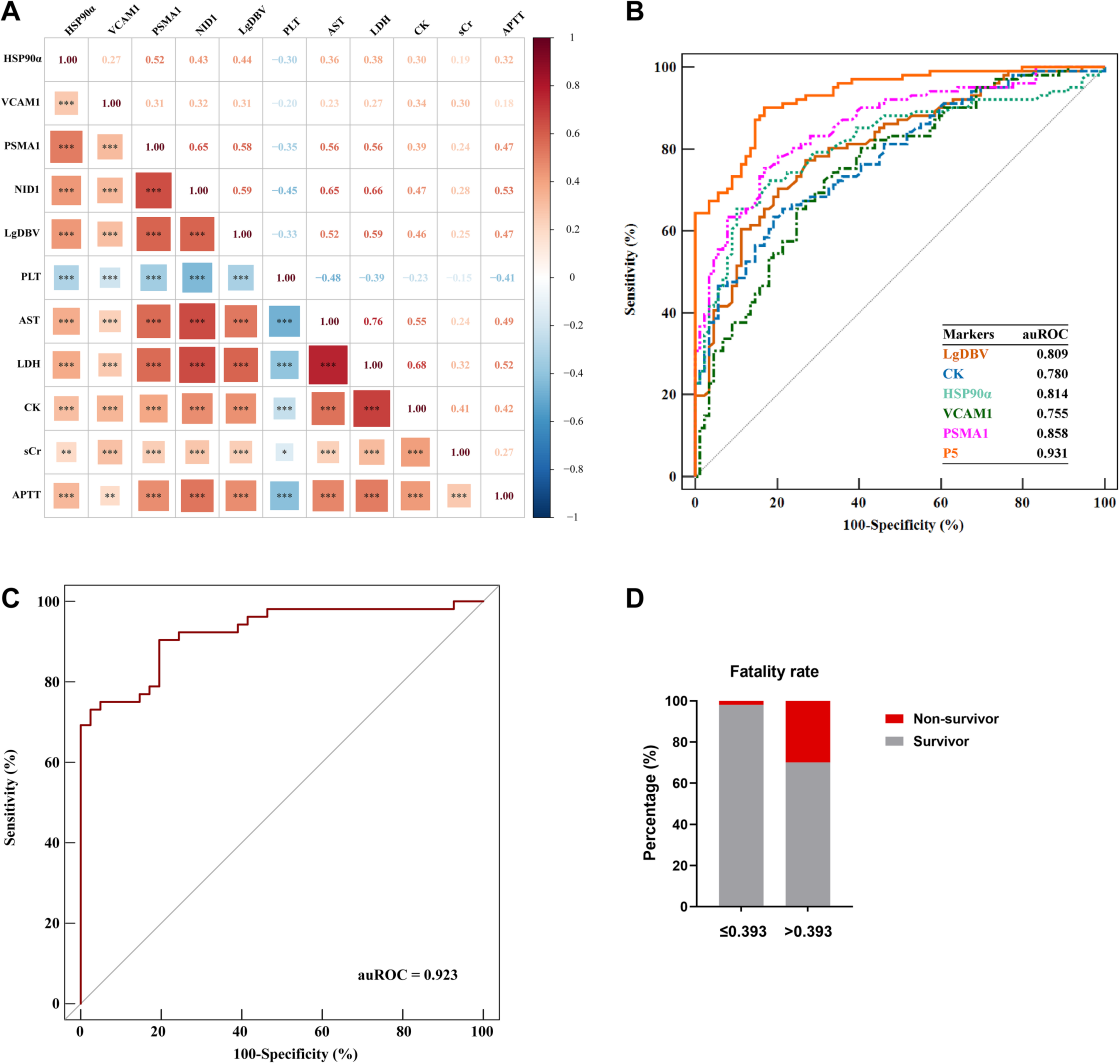
Development of the P5 model in the early stage for prediction of severe illness. **(A)** Spearman correlation heatmap showed that HSP90α, VCAM1, PSMA1, and NID1 were significantly associated with the severity-related indicators. Red represents positive correlations; blue represents negative correlations; and color depth and square size represent the intensity of the correlation. Significant correlations are marked by asterisks; *, **, and *** represent *p* < 0.05, *p* < 0.01, and *p* < 0.001, respectively. **(B)** Comparison of the predictive efficiency of P5, LgDBV, CK, HSP90α, VCAM1, and PSMA1 for identifying severe illness in SFTS patients using ROC plots in the evaluation group (Cohort 2). **(C)** Validation of the prediction efficacy of P5 using ROC curve analysis in the validation cohort (Cohort 3). **(D)** Comparison between the fatality rates of patients with P5 levels greater than 0.393 (*n* = 177) and patients with P5 levels lower than 0.393 (*n* = 106); red and grey represent the rate of non-survivor and survivor, respectively. Data were collected at the time of initial diagnosis within the early stage in each patient. Data are presented as medians with interquartile ranges. *p*-values were obtained by the Mann–Whitney test.

Plasma samples in early-stage and clinical parameters were further obtained from 283 SFTS patients, then, we used ELISA to detect the candidate protein concentrations. A correlation analysis revealed that HSP90α, VCAM1, PSMA1, and NID1 were correlated with the levels of severity-related indicators such as PLT, APTT, AST, LDH, CK, and sCr (Figure 4A), suggesting these proteins can serve as markers reflecting the multi-organ damage in patients with SFTS.

These results indicated those four candidate proteins had a close association with the disease progression, and higher concentrations of those protein markers indicate a more severe state of illness.

### Early prediction model for severe SFTS patients

In order to develop an early prediction model for severe illness, protein markers were detected in plasma by ELISA and clinical data were collected in the early stage from 190 patients with SFTS from June 2015 to February 2021 (Cohort 2). The clinical characteristics and the concentration of four protein markers were summarized in Table 3. We performed univariate logistic regression analysis to identify variables with *p*-values less than 0.001. Subsequently, we constructed an optimal model using the stepwise method, where a variable was included if its *p*-value was less than 0.05 and removed if its *p*-value exceeded 0.1. This process resulted in a model comprised of five predictor variables including LgDBV, CK, HSP90α, VCAM1, and PSMA1 (P5, all with auROC >0.75), named LCHVP. The model can be expressed as follows:


$$\begin{array}{l} Ln\;\left(P5/\left(1-P5\right)\right)=-8.732+0.793^{*}\mathrm{LgDBV}+0.001^{*}CK\\ \;+0.011^{*}HSP90\alpha +0.001^{*}\mathrm{VCAM1}+\mathrm{PSMA1} \end{array}$$

**Table 3:** Comparisons of the clinical characteristics and protein markers between mild and severe patients with SFTS.

Variable	Mild (*n* = 89)	Severe (*n* = 101)	Univariate analysis *p*-value	Multivariate logistic regression	
				OR (95% CI)	*p*-value^a^
Age (year)	59.0 ± 13.7	64.5 ± 10.5	**0.003**	—	—
Male, *n* (%)	42 (47.2)	47 (52.8)	0.388		
Hypertension	18 (20.2)	37 (36.6)	**0.014**	—	—
Diabetes	6 (6.7)	9 (8.9)	0.580		
Laboratory parameters on admission					
LgDBV (copies/mL)	5.3 ± 1.0	6.7 ± 1.3	**<0.001**	2.40 (1.32–4.37)	**0.004**
WBC (×10^9^/L)	2.0 (1.6, 3.4)	2.2 (1.5, 3.6)	0.546		
Neutrophil (×10^9^/L)	1.9 (1.2, 4.7)	2.1 (1.2, 4.0)	0.933		
Lymphocyte (×10^9^/L)	0.6 (0.4, 0.8)	0.6 (0.4, 1.0)	0.513		
Monocyte (×10^9^/L)	0.1 (0.1, 0.2)	0.1 (0.1, 0.2)	0.821		
PLT (×10^9^/L)	65 (47, 87)	40 (28, 58)	**<0.001**	0.98 (0.97–1.00)	0.069
Hemoglobin (g/L)	145 (131, 154)	147 (133, 159)	0.581		
AST (U/L)	105 (78, 168)	351 (195, 603)	**<0.001**	1.00 (1.00–1.01)	0.061
LDH (U/L)	488 (368, 762)	1,121 (658, 2,164)	**<0.001**	—	—
CK (U/L)	257 (104, 490)	907 (323, 1703)	**<0.001**	1.00 (1.00–1.01)	**0.008**
Albumin (g/L)	36 (33, 38)	31 (28, 35)	**<0.001**	0.91 (0.88–1.02)	0.088
sC (μmol/L)	66 (54, 81)	74 (56, 111)	**0.004**		
BUN (mmol/L)	5.0 (3.6, 6.6)	6.2 (4.3, 10.3)	0.552		
APTT (s)	39 (35, 46)	47 (41, 58)	**<0.001**	—	—
Protein markers					
HSP90α (ng/mL)	42 (19, 70)	118 (67, 214)	**<0.001**	1.01 (1.00–1.02)	**0.029**
VCAM1 (ng/mL)	592 (312, 1,038)	1,211 (756, 2,308)	**<0.001**	1.00 (1.00–1.00)	**0.002**
PSMA1 (ng/mL)	0.9 (0.7, 1.4)	2.4 (1.5, 3.8)	**<0.001**	2.30 (1.18–4.46)	**0.014**
NID1 (ng/mL)	3,566 ± 1,326	6,473 ± 2,587	**<0.001**	—	—

SFTS, severe fever with thrombocytopenia syndrome; DBV, dabie bandavirus; WBC, white blood cell; PLT, platelet; AST, aspartate aminotransferase; LDH, lactate dehydrogenase; CK, creatine kinase; sCr, serum creatinine; BUN, blood urea nitrogen; APTT, activated partial thromboplastin time. Lg, logarithm of 10; OR, odds ratio; CI, confidence interval. Data are presented as the mean ± standard deviation, median (*p*_25_, *p*_75_), and proportion. The bold value indicates *p* < 0.05.

a Multivariable analysis included all significant (*p* < 0.001) variables using a stepwise method.

ROC curve analysis revealed that P5 had the highest auROC of 0.931 (95% CI, 0.885, 0.963) at the optimal cut-off value of 0.393. The sensitivity and specificity of P5 for predicting severe SFTS were 90.1 and 83.2%, respectively (Figure 4B and Table 4). Subsequently, we performed external validation in Cohort 3, which consisted of 93 patients from March 2022 to November 2022. There were no differences in age, death rate or any laboratory values between Cohort 2 and Cohort 3 (Table 5), and the results showed that the auROC in the validation group was 0.923 (Figure 4C). Moreover, we found that patients with a P5 value greater than 0.393 had a fatality rate of 29.9% (95% CI, 23.3–37.3%), which was significantly higher than the fatality rate in patients with lower P5 levels [1.9% (95% CI, 0.2–6.5%), *p* <0.001; Figure 4D].

**Table 4:** The five-biomarker combination (P5) for classifying SFTS patients and predicting progression from mild to severe conditions.

Markers	ROC analysis					Multivariate logistic regression	
	Cut-off value	auROC (95% CI)	Sensitivity (%)	Specificity (%)	*p*-value (vs. LgDBV)	Coefficient	*p*-value
LgDBV (copies/mL)	5.88	0.809 (0.746–0.862)	77.2	73.0	—	0.793	**0.003**
CK (U/L)	533	0.780 (0.715–0.837)	63.4	80.9	0.477	0.001	**0.004**
HSP90α (ng/mL)	97	0.814 (0.751–0.867)	65.4	89.9	0.907	0.011	**0.013**
VCAM1 (ng/mL)	822	0.755 (0.688–0.814)	72.3	68.5	0.250	0.001	**0.010**
PSMA1 (ng/mL)	1.45	0.858 (0.800–0.904)	75.3	83.2	0.161	1.000	**0.001**
Constant	—	—	—	—	—	−8.732	—
P5	0.393	0.931 (0.885–0.963)	90.1	83.2	**<0.001**	—	—

Protein markers description		
Gene	Protein	Function
HSP90α	Heat shock protein 90α	Activation of innate immune response, cellular response to virus
VCAM1	Vascular cell adhesion protein 1	Cell adhesion/differentiation, inflammatory response, response to extracellular stimulus
PSMA1	Proteasome subunit alpha type 1	Immune system process, regulation of inflammatory response to antigenic stimulus, proteasomal protein catabolic process

SFTS, severe fever with thrombocytopenia syndrome; DBV, dabie bandavirus; CK, creatine kinase. Lg, logarithm of 10; ROC, receiver operating characteristic curve; auROC, area under the ROC; CI, confidence interval. The bold value indicates *p* < 0.05.

**Table 5:** Comparison of baseline clinical characteristics in SFTS patients between Cohort 2 and Cohort 3.

Characteristic	All patients	Cohort 2	Cohort 3	*p*-value
Number of patients	283	190	93	—
Age (year)	61.9 ± 11.6	61.9 ± 12.4	62.0 ± 9.8	0.964
Male, *n* (%)	143 (50.5)	96 (50.5)	47 (50.5)	1.000
Hypertension	76 (26.9)	55 (28.9)	21 (22.6)	0.256
Diabetes	26 (9.2)	15 (7.9)	11 (11.8)	0.282
Complications, *n* (%)				
Encephalitis	74 (26.1)	43 (22.6)	31 (33.3)	0.054
Multiple organ failure	39 (13.8)	25 (13.2)	14 (15.1)	0.664
Severe hemorrhage	36 (12.7)	23 (12.1)	13 (14.0)	0.657
Severe infection	13 (8.5)	8 (4.2)	5 (5.4)	0.890
Severe case, *n* (%)	153 (54.1)	101 (53.2)	52 (50.3)	0.662
28 days death, *n* (%)	55 (19.4)	38 (20.0)	17 (18.3)	0.731
Laboratory parameters on admission				
LgDBV (copies/mL)	6.1 ± 1.3	6.0 ± 1.4	6.2 ± 1.2	0.320
WBC (×10^9^/L)	2.2 (1.5, 3.3)	2.0 (1.5, 3.1)	2.4 (1.7, 3.5)	0.095
Neutrophils (×10^9^/L)	1.3 (0.9, 2.1)	1.2 (0.9, 2.1)	1.4 (1.1, 2.0)	0.053
Lymphocytes (×10^9^/L)	0.6 (0.4, 1.0)	0.6 (0.4, 0.9)	0.6 (0.4, 1.1)	0.303
Monocytes (×10^9^/L)	0.1 (0.1, 0.2)	0.1 (0.1, 0.2)	0.1 (0.1, 0.3)	0.589
PLT (×10^9^/L)	53 (36, 75)	50 (34, 76)	58 (41, 73)	0.078
ALT (U/L)	71 (43, 134)	78 (45, 135)	64 (38, 132)	0.159
AST (U/L)	181 (91, 378)	192 (96, 417)	161 (81, 324)	0.075
LDH (U/L)	691 (417, 1,208)	742 (455, 1,298)	571 (356, 1,148)	0.052
CK (U/L)	445 (173, 1,071)	445 (180, 1,072)	382 (158, 1,024)	0.600
ALB (g/L)	34 (30, 37)	34 (30, 37)	34 (30, 38)	0.648
sCr (μmol/L)	68 (55, 88)	68 (56, 88)	66 (54, 86)	0.970
BUN (mmol/L)	5.5 (4.1, 7.9)	5.3 (4.1, 8.4)	5.6 (4.0, 7.2)	0.315

SFTS, severe fever with thrombocytopenia syndrome; DBV, dabie bandavirus; WBC, white blood cell; PLT, Platelet; ALT, alanine aminotransferase; AST, aspartate aminotransferase; LDH, lactate dehydrogenase; CK, creatine kinase; ALB, albumin; sCr, serum creatinine; BUN, blood urea nitrogen; Lg, logarithm of 10. Data are presented as the mean ± standard deviation, median (*p*_25_, *p*_75_), and proportion.

## Discussion

SFTS was considered one of the most challenging conditions in the realm of newly emerging infectious diseases with high mortality rate. It is of high priority to identify biomarkers that can monitor and predict disease progression. Therefore, our study recruited a large sample of SFTS patients to validate the potential biomarkers discovered by plasma DIA proteomics and observed its dynamic changes at different stages of the disease course. Finally, we observed that elevated levels of HSP90α, VCAM1, PSMA1, and NID1 were positively associated with multi-organ dysfunction and disease deterioration. More importantly, the prediction model of P5 developed based on plasma protein biomarkers and clinic data had an effective predictive efficacy for severe illness. SFTS patients in the early stage with P5 score greater than 0.393 had a high risk of death within 28-days after onset. Our findings provide clinicians with an assessment method to early identify SFTS patients at high risk of developing severe or fatal outcomes, enabling timely guidance for optimal clinical management of SFTS and improving its prognosis.

The previously identified biomarkers and risk factors for the progress of SFTS were almost all clinical or laboratory indicators, and each single biomarker could only indicate damage to one or two specific organs. However, SFTS is characterized by damage in multiple tissues and organs. Thus, although many indicators have been discovered to be associated with severe conditions, such as PLT, LDH, CK, or encephalopathy (Yu, 2018; Wang et al., 2019; Zhao et al., 2022), it still lacks indicators that could indicate multiple tissue damage together. Here, we identified the alterations of plasma proteins including HSP90α, VCAM1, PSMA1, and NID1 well correspond to the severity and progress of patients with SFTS. Those protein biomarkers indeed reflected multiple organ impairment including coagulation disorder, and myocardial, liver, or renal, might serve as valuable predictors for severe patients with SFTS.

It is well known that DBV-mediated excessive inflammation of the host played an important role in the progression of the disease (Sun et al., 2012; Liu Q. et al., 2014). The host’s intense inflammatory response after DBV infection leads to extensive pathological lesions in multiple organs, ultimately causing extreme abnormalities in biochemical parameters and clinical symptoms in SFTS patients (Ding et al., 2014). However, the identified biomarkers initially elevated in plasma and are closely associated with this inflammatory damage in hosts after DBV infection. Among them, HSP90α was demonstrated to participate in the activation and regulation of innate immune responses in pathogen infections (Calderwood et al., 2016; Hoter et al., 2018). It also has been reported that HSP90α could be maintaining the stability of rubella virus proteins that facilitate viral replication *in vivo* (Sakata et al., 2019). Similarly, another member of the heat shock protein family has also been associated with the severity of SFTS in the early stage (Ding et al., 2014). For VCAM1, a cell adhesion glycoprotein primarily expressed on the surface of vascular endothelial cells, acts as a critical regulator of leukocyte adhesion to the endothelium and transendothelial migration (Ou et al., 2008). Previous studies have revealed both DBV virus and host cytokines can induce vascular endothelial injury and barrier dysfunction (Li et al., 2017; Li X. K. et al., 2018). Thus, elevated VCAM1 might indicate excessive immune responses and vascular endothelial injury in patients with SFTS. NID1 is a sulfated glycoprotein that is widely distributed in basement membranes. It plays a crucial role in stabilizing the basement membrane and inducing immune cells chemotaxis (Timpl and Brown, 1996). Certain viruses, such as cytomegalovirus, have been observed to impact the structure and function of the cell basement membrane by altering NID1 expression to facilitate virus dissemination and disease progress (Kuan et al., 2022). Additionally, multiple RNA viruses have been found to modulate the ubiquitin-proteasome system in various mechanisms, including immune evasion, virus entry and release, transcriptional regulation, and inhibition of cell apoptosis (Choi et al., 2013). PSMA1, a subunit of the 20S proteasome involved in the proteasome metabolic pathway, is probably involved in the mechanism mentioned above. Therefore, those proteins, which are primarily involved in immunity, not only can serve as effective predictors in severe patients with SFTS but also might provide insights into potential severe-related pathological mechanisms.

Consistent with previous reports (Gai et al., 2012; Wang Y. et al., 2022), viral load was also the most vital predictor for severe illness in our study. High viremia triggers the activation of immune cells, leading to excessive cytokine storm, resulting in immune imbalance and extensive organ damage that promotes disease progression (Kwon et al., 2018; Yang et al., 2022). Our study also found that the myocardial cell injury parameter CK was an important indicator for predicting severe SFTS. In the multiple organ dysfunction caused by DBV infection, the myocardial serves as the primary target tissue, early deterioration of the cardiac usually indicates a more severe condition, which has been confirmed in many previous studies (Cui et al., 2012; Cui et al., 2015; Yu, 2018; He et al., 2021; Wang et al., 2021; Song et al., 2022). The possible reasons were linked to dramatic inflammatory damage caused by the pathogens. Additionally, the direct replication of the virus in organs might be another contributing factor. DBV virus replication in the heart was observed in pathological tissues of deceased patients with SFTS, as well as in animal models (Hiraki et al., 2014; Liu Y. et al., 2014).

It should be noted that our prediction model was developed based on proteomic and clinical parameters at the time of early stage, some clinical symptoms that cannot be fully confirmed to occur at this time were excluded from this study. However, although the patients were not all admitted at an early stage in actual clinical practice, the correlation of protein markers alternation with the change of the state of illness, we believe that sequential measurements of these protein biomarkers in SFTS patients also provide molecular trajectories toward convalescence or deterioration, thus to broaden the utilization of the current early-stage-based model. We also emphasize that our study has produced a wealth of potential biomarkers highly correlated with SFTS, only a few were included in the final model. However, we should not underestimate the potential value and biological effects of the rest candidates that were not integrated into our predictive model. We anticipate that further exploratory basic research based on proteomics by our team will thoroughly reveal the specific molecular mechanisms of these protein markers. Furthermore, our study sample was exclusively from a single center in a tertiary hospital and may not be fully representative of the overall population. Therefore, although our study systematically revealed the proteomic alternations and developed an early effective prediction model for severe cases, a multicenter approach to subject recruitment for validating the value of the model would be preferable.

In summary, the proteomics discovered in our research sheds light on the potential pathogenesis of patients with SFTS. The prediction model developed based on protein biomarkers has demonstrated outstanding predictive performance in predicting the occurrence of severe illness. This model aids physicians in the early identification of high-risk patients and merits more attention on them, and in formulating optimal clinical strategies to avoid adverse outcomes.

## Data Availability

The datasets presented in this study can be found in online repositories. The names of the repository/repositories and accession number(s) can be found in the article/Supplementary material.
