# Supplementary material for: Identification of early prediction biomarkers of severity in patients with severe fever with thrombocytopenia syndrome based on plasma proteomics
Source: Front Microbiol. 2025 Feb 5;16:1514388. doi: 10.3389/fmicb.2025.1514388 (PMC11836002; doi:10.3389/fmicb.2025.1514388)
Supplement: Supplementary file 1 [file Data_Sheet_1.docx]

**DIA Methods**

1. **Protein extraction**

1) Take 20ul of each sample and mix it into one sample. Add 200ul of each sample to the pre cleaned beans with high abundance, and mix it for 2h at RT;

2) 4 ℃, 5000rpm/min, centrifugation for 5min, pour out the supernatant, and wash beans with wash buffer for three times;

3) Add 200ul 1% TFA, shake and mix for 10min, centrifugate the supernatant, and repeat twice;

4) Combine the supernatant twice, freeze dry, and then use 50 ul 8M UA redissolution;

5) Take 5ul of the remaining samples and mixed samples respectively, add 120ul of 8M UA, vortex and mix evenly. The protein concentration was determined using Bradford method，rest was frozen to -80℃.

1. **Protein digestion and desalination and Library construction**

A 100µg aliquot of extracted proteins from each sample was then subjected to reduction. Adding 200 mM dithiothreitol (DTT) solution and incubating at 37 °C for 1 h. The sample was diluted 4 times by adding 25 mM ammonium bicarbonate (ABC) buffer, then adding trypsin (trypsin: protein =1:50) and incubating at 37℃ overnight.

The next day, adding 50μL 0.1% FA to terminate the digestion, take 100μl 100% ACN to wash the C18 column, and centrifuge at 1200rpm for 3min. Wash the column once with 100 μl of 0.1% FA and centrifuge at 1200 rpm for 3 min. Replace the EP tube, add the sample, and centrifuge at 1200rpm for 3min. Wash the column twice with 100 μl of 0.1% FA and centrifuge at 1200 rpm for 3 min. Wash once with 100 μl of pH 10 water. Replace the EP tube and elute with 70% ACN. The eluents of each sample were combined and lyophilized. Store at -80°C until loading.

The sample peptides were fractionated using a C18 column (Waters BEH C18, 4.6×250 mm, 5 µm) on a Rigol L3000 HPLC operating at 1 mL/min, with the column oven being set to 50 ℃. Mobile phases A [2% acetonitrile (ACN), adjusted pH 10.0 using ammonium hydroxide] and B (98% ACN, adjusted pH 10.0 using ammonium hydroxide) were used to develop a gradient elution. The solvent gradient was set as follows: 5% B, 0 min; 5–8% B, 5 min; 8–18% B, 35 min; 18–32% B, 22 min; 32–95% B, 2 min; 90% B, 4 min; 95–5% B, 4 min. The eluates were monitored at UV 214 nm, collected for a tube per minute, and finally merged into 6 fractions. The 6 fractions peptides were dried in a vacuum. The sample peptides and the 6 fractions of peptides were reconstituted in 0.1% (v/v) formic acid (FA) in water. Then, 0.2 µL of standard peptides (iRT kit, Biognosys) were added into the peptide sample for subsequent analyses. For transition library construction, shotgun proteomics analyses were performed using an EASY-nLCTM 1200 UHPLC system coupled with an Orbitrap Exploris™480 mass spectrometer (Thermo Fisher Scientific) operating in the data-dependent acquisition (DDA) mode.

1. **LC-MS/MS Analysis**

For spectral library generation, samples were fractionated using a high pH reversed-phase fractionator as previously described and measured in DDA mode. Nanoflow LC-MS/MS analysis of tryptic peptides was conducted on the Thermo Scientific Orbitrap Exploris™480 platform coupled to an EASY nLC 1200 ultra-high pressure system (Thermo Fisher Scientific) via a nano-electrospray ion source.500 ng of peptides were loaded on a 25 cm column (150 μm inner diameter, packed using ReproSil-Pur C18-AQ 1.9- µm silica beads; Beijing Qinglian Biotech Co.,Ltd, Beijing, China). Peptides were separated using a gradient from 6% to 12% B in 8 min, then12% to 30 % B in 55 min and stepped up to 40% in 12 min followed by a 15 min wash at 95% B at 600 nl per minute where solvent A was 0.1% formic acid in water and solvent B was 80% ACN and 0.1% formic acid in water. The total duration of the run was 90 min. Briefly, the mass spectrometer was operated in “top-40” data-dependent mode, collecting MS spectra in the Orbitrap mass analyzer (60,000 resolution, 350–1200 m/z range) with an automatic gain control (AGC) target of Custom and a maximum ion injection time of 50 ms. The most intense ions from the full scan were isolated with an isolation width of 1.6 m/z. Following higher-energy collisional dissociation (HCD) with a normalized collision energy (NCE) of 30, MS/MS spectra were collected in the Orbitrap (15,000 resolution) with an AGC target of Custom and a maximum ion injection time of 22 ms. Precursor dynamic exclusion was enabled with a duration of 16 s.

The data-independent acquisition (DIA) scan mode was used for tissue samples. For DIA acquisition, the MS1 resolution was set to 120,000 (at 200 m/z), and the MS2 resolution was set to 15,000 (at 200 m/z). The m/z range covered from 350 to 1,500 m/z and was separated into 42 acquisition windows (arying isolation windows from 14 m/z to 312 m/z). The full scan AGC target was set to Custom. DIA settings included NCE of 33%, a target value of Custom, and an automatic maximum injection time was set to automatic to allow the MS to continuously operate in the parallel ion filling and detection mode.

1. **Data analysis**

**4.1 The identification and quantitation of protein**

Mass spectrometry data processing The MS data of the fractionated pools (DDA MS data, 6 fractions) and the single-shot subject samples (DIA MS data) were used to generate a hybrid library in the Spectronaut software (Biognosys, version 15.7.220308.50606). The hybrid spectral library was used to search the MS data of the single-shot samples in the Spectronaut software, for final protein identification and quantitation. All searches were performed against the human UniProt reference proteome of canonical and isoform sequences with 20,373 entries downloaded in October 2021. Searches used carbamidomethylation as fixed modification and acetylation of the protein N-terminus, oxidation of methionines as variable modifications. Default settings were used for other parameters. In brief, a trypsin/P proteolytic cleavage rule was used, permitting a maximum of two miscleavages and a peptide length of 7–52 amino acids. Protein intensities were normalized using the “Local Normalization” algorithm in Spectronaut based on a local regression model (Callister et al, 2006). Spectral library generation stipulated a minimum of three fragments per peptide, and maximally, the six best fragments were included. A protein and precursor FDR of 1% were used and protein quantities were reported in samples only if the protein passed the filter.
